# First person – Yutian Li

**DOI:** 10.1242/bio.062938

**Published:** 2026-10-23

## Abstract

First Person is a series of interviews with the first authors of a selection of papers published in Biology Open, helping researchers promote themselves alongside their papers. Yutian Li is first author on ‘ Early injury responses to nutrient treatment in adult *Drosophila* limb’, published in BiO. Yutian conducted the research described in this article while a graduate student in Lea Goentoro's lab at the Division of Biology and Biological Engineering, California Institute of Technology (Caltech), Pasadena, USA. She is now a volunteer researcher in Lea Goentoro's lab, investigating how cells and tissues interpret environmental and physiological signals, and how these signals drive lasting changes in cell state.

**Figure:**
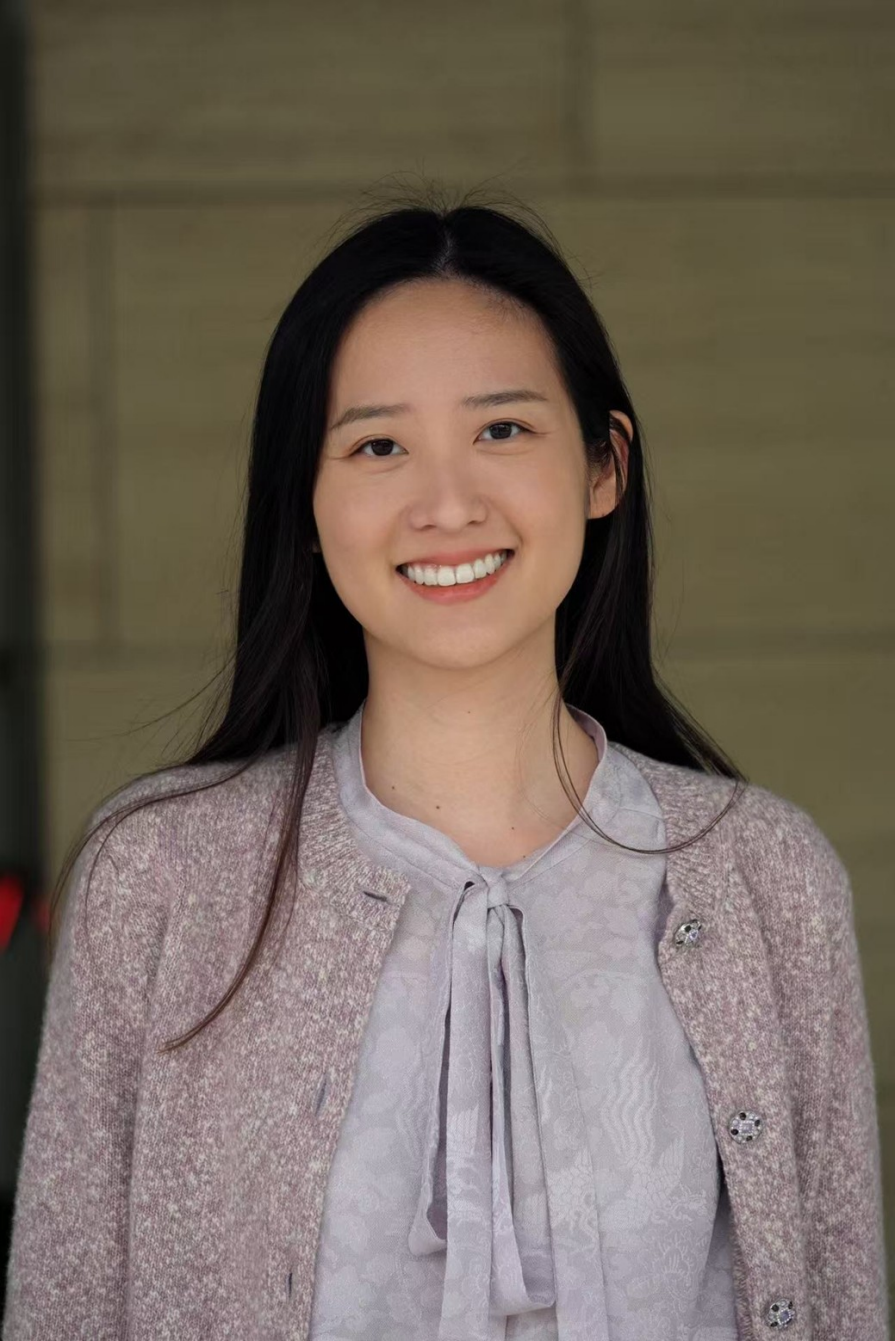
Yutian Li

**Describe your scientific journey and your current research focus**

My scientific journey began at UC San Diego, USA, where I studied how yeast cells encode memories of environmental stress through dynamic signaling. During my PhD at Caltech, I shifted to regeneration, asking whether nutrient treatment could alter the injury response of an adult *Drosophila* limb that normally does not regrow. My PhD work focused on characterizing the early cellular and tissue changes associated with this response.

**Who or what inspired you to become a scientist?**

I was first drawn to research through Dr Hao's molecular biology course at UC San Diego. I enjoyed learning how molecular biology could be worked out through experiments – how a change in a gene might affect the RNA or protein produced, or how a gel or other experimental readout could reveal what was happening in a cell. That experience led me to join his lab, where I had my first experience doing research.

**How would you explain the main finding of your paper?**

Normally, adult fruit flies do not regrow injured limbs, but we found that giving the flies a particular combination of nutrients changes the response to injury: the wound heals differently, more of the injured tissue stays alive, and some flies show partial limb regrowth. We also found distinct muscle and epithelial cell states in the surviving tissue, characterised by higher expression of genes involved in cell division and tissue growth.

**What are the potential implications of this finding for your field of research?**

Our findings suggest that nutrient availability can alter early responses to injury in adult tissues with limited regenerative capacity, including improved tissue survival and distinct cellular states associated with regeneration. While regeneration of a complete organ remains a longer-term goal, preserving injured tissue could be a valuable intermediate outcome, and these early tissue and cellular changes may provide useful markers for further study of how regrowth begins.

**Figure:**
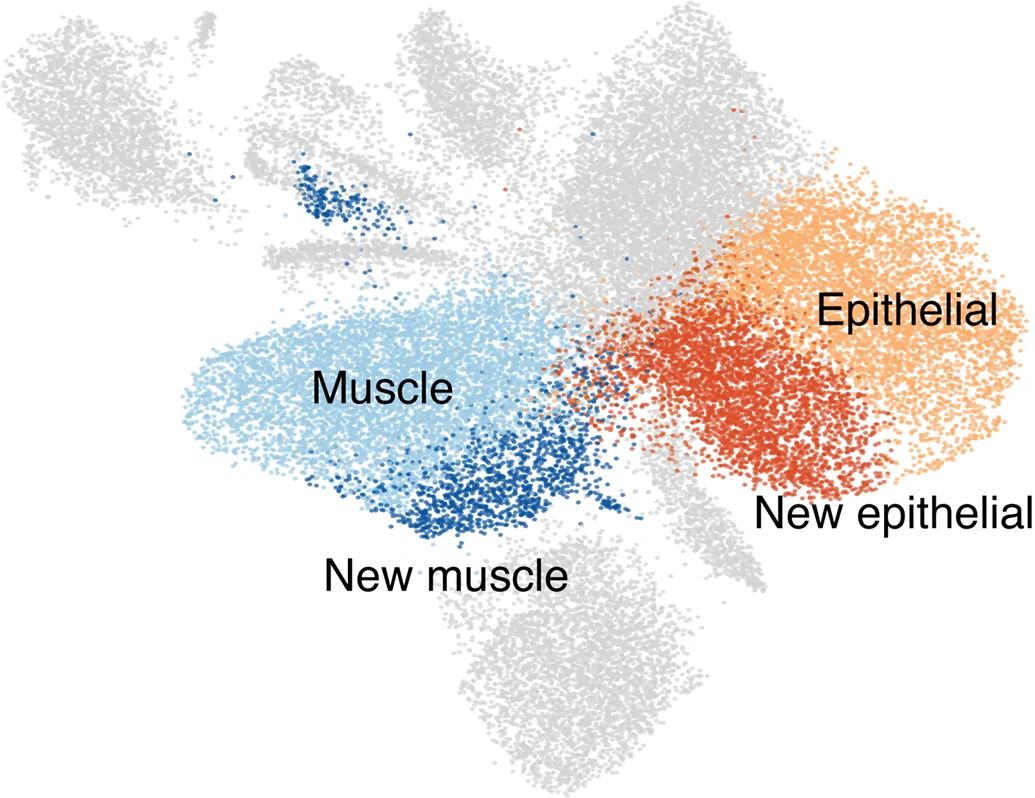
Single-nucleus RNA-sequencing reveals distinct muscle and epithelial cell states in surviving adult *Drosophila* limb tissue.

**Which part of this research project was the most rewarding?**

The most rewarding moment was when the single-nucleus RNA-sequencing data revealed distinct muscle and epithelial cell states in the surviving tissue that were not present in normal limbs. These cells showed higher expression of genes involved in cell division and tissue growth, including genes associated with regeneration in larval tissues. It was exciting to see a tissue-level phenotype resolve into specific cellular states.

**What do you enjoy most about being an early-career researcher?**

I enjoy having the freedom to explore new questions and learn new approaches along the way. At this stage, I am still figuring out what kinds of problems I want to pursue long term, and I like being able to move between different systems and methods.

**What piece of advice would you give to the next generation of researchers?**

Pay attention to the results you did not set out to find. Some of the most interesting directions in my PhD came from observations that were not part of the original plan. Even results that seem disappointing at first can be informative if they make you look at the problem in a different way.

**What's next for you?**

I recently completed my PhD and am now looking for my next research opportunity. I hope to continue using single-cell approaches to study cell states in the context of human disease, particularly in cancer.

## References

[BIO062938C1] Li, Y., del Rio Salgado, J., Bates, G., Condiotte, Z. J., Caskey, M., Dimayuga, C. M., Williams, B., Hallgrimsdottir, I., Pachter, L. and Goentoro, L. (2026). Early injury responses to nutrient treatment in adult *Drosophila* limb. *Biol. Open* 15, bio062737. 10.1242/bio.062737 PMC13730125 42802989

